# Atrial Fibrillation and Pacing Algorithms

**Published:** 2008-08-01

**Authors:** Paolo Terranova, Barbara Severgnini, Paolo Valli, Simonetta Dell'Orto, Enrico Maria Greco

**Affiliations:** * U.O. Cardiologia e UTIC, Azienda Ospedaliera "S. Paolo" - Polo Universitario, Department of Medicine, Surgery and Odontoiatry, University of Milan, Italy; † U. O. di Cardiologia, Presidio Ospedaliero "Causa Pia Ospedaliera Uboldo", Cernusco sul Naviglio, Azienda Ospedaliera di Melegnano, Milano, Italy

Manuscript retracted by first author with apologies for erroneous submission as it was quite similar to another article published by another group of authors in another journal earlier. The author mentioned that it was a non voluntary mistake and requested immediate erasal of the paper. The author has also informed that the co-authors had no role in this error.

